# Antibiotic-resistant status and pathogenic clonal complex of canine *Streptococcus canis*-associated deep pyoderma

**DOI:** 10.1186/s12917-022-03482-3

**Published:** 2022-11-09

**Authors:** Ichiro Imanishi, Keita Iyori, Akira Také, Ryota Asahina, Manami Tsunoi, Ryuji Hirano, Jumpei Uchiyama, Yoichi Toyoda, Yoshihiko Sakaguchi, Shunji Hayashi

**Affiliations:** 1 Department of Microbiology, Kitasato University School of Medicine, 1-15-1 Kitasato, Minami-ku, Sagamihara-shi, Kanagawa Japan; 2 Dermatological and Laboratory Service for Animals, Vet Derm Tokyo, 910 Shobusawa, Fujisawa, Kanagawa 252-0823 Japan; 3 Department of Dermatology, Graduate School of Medicine, Kyoto University, 54 Shogoin-Kawahara-cho, Sakyo-ku, Kyoto, 6068501 Japan; 4 Kyoto Animal Medical Center, 550-4 Bishamon-cho, Nakagyo-ku, Kyoto, 6040984 Japan; 5 Research Center for Medical Sciences, The Jikei University School of Medicine, 3-25-8, Nishi-Shimbashi, Minato-ku, Tokyo, 105-8461 Japan; 6 Ukyo Animal Hospital, 12-2 Uzumasa-Kyonomichi-cho, Ukyo-ku, Kyoto, 6168181 Japan; 7 Department of Bacteriology, Graduate School of Medicine Dentistry and Pharmaceutical Sciences, 1-1-1, Tsushima-naka, Kita-ku, Okayama University, Okayama, 7008530 Japan

**Keywords:** Dogs, *Streptococcus*, Antibiotic resistance, Multilocus sequence typing, Pyoderma, Oral cavity, Beta-lactamase, Opportunistic infections

## Abstract

**Background:**

*Streptococcus canis* causes deep pyoderma in canines, which raises concerns about the risk of isolates from lesions acquiring an antibiotic-resistant phenotype. It is necessary to identify effective antibiotics and the characteristics of the pathogenic cluster for *S. canis*-associated deep pyoderma.

**Results:**

The signalment, molecular typing, and antibiotic-resistant status of *S. canis* isolated from deep pyoderma lesions (27 strains) and oral cavities (26 strains) were analyzed. Older dogs tended to have *S. canis*-associated deep pyoderma (15 of 27 dogs over 10 years old). Veterinarians chose quinolones for 10/16 cases (63%), even though the rate of quinolone-resistant strains of *S. canis* is 38–59%. Although 70% of the strains showed resistance to three or more antibiotic classes (37/53), 94% (50/53) strains showed sensitivity for penicillins. We also identified β-lactamase activity among penicillin-resistant strains of *S. canis*. Clonal complex 13 (CC13) was detected only in lesions and formed independent clusters in the phylogenetic tree. One strain of CC13 was resistant to the anti-methicillin-resistant *Staphylococcus aureus* drugs, vancomycin and linezolid.

**Conclusion:**

Although antibiotic-resistant strains of *S. canis* are isolated at a high rate, they can currently be treated with β-lactamase-inhibiting penicillins. CC13 may be a pathogenic cluster with high levels of antibiotics resistance.

**Supplementary Information:**

The online version contains supplementary material available at 10.1186/s12917-022-03482-3.

## Background

Canine deep pyoderma is a bacterial infection that affects tissues deeper than the epidermis, such as cellulitis and furunculosis [1]. The development of drug resistance by pathogenic bacteria is a critical problem for the treatment of pyoderma [2]. The current recommendation for deep pyoderma is to treat for a minimum of 6–10 weeks with a maximum dose of systemic antibiotics [1]. Appropriate antibiotic agents should be used depending on the pathogenic bacterial species and their drug-resistant status because long-term systemic antibiotic administration can cause resistant strains [3].

*Streptococcus canis* is known to be one of the causative gram-positive cocci of deep pyoderma in dogs and humans [1, 4]. *S. canis*-associated deep pyoderma sometimes causes a potentially fatal prognosis [5]. It is also essential as a zoonotic pathogen because keeping dogs with *S. canis* puts their owners at risk of developing deep pyoderma [6]. However, scant signalment information on *S. canis*-associated deep pyoderma has been reported to date, and it is not known which dogs are more likely to develop the disease*.*

*S. canis* is a β-hemolytic Lancefield group G streptococcus, which colonizes the skin, the oral cavity, the upper respiratory tract, and the reproductive tract of dogs [7, 8]. *S. canis*-associated deep pyoderma is thought to be an opportunistic infection, with resident bacteria infecting the destroyed skin [1, 9]. Streptococci dominate the oral cavity of dog*s,* and *S. canis* is endemic [10, 11]. Because dogs tend to lick their wounds when their skin is broken, opportunistic infection from the oral cavity is suspected, but this hypothesis has not yet been tested.

Unlike staphylococci, most streptococci are not penicillin-resistant; therefore, penicillins are used as first-line therapy in animals and humans [12, 13]. However, the use of penicillins to treat infections with streptococci is increasing in Japan, and β-lactam-resistant strains of streptococci are being reported with increasing frequency [14, 15]. *S. canis* has been reported to be resistant to quinolones (resistance rate, 7.0%) and tetracyclines (resistance rate, 5.6–39.7%), although there have been no reports of resistance to β-lactam drugs [14, 16–18]. The strains used in those analyses of the resistance status of *S. canis* in dogs were a mixture of strains of various origins, including earwax-derived, oral-derived, and lesion-derived strains, and to our knowledge, no detailed information on the antibiotic resistance status of *S. canis* from canine deep pyoderma is available to date.

In the present study, we analyzed the signalment of *S. canis* isolated from deep pyoderma lesions and the oral cavity. We compared the molecular typing and antibiotic resistance of the strains to determine the essential clinical features of *S. canis*-associated deep pyoderma and clarify whether it is an opportunistic infection. We also aimed to clarify which antibiotic agents should be used for patients with deep pyoderma associated with *S. canis.*

## Results

### Clinical features of dogs with *S. canis*-associated deep pyoderma

Gram-positive cocci were detected from lesion contents from 546 of 719 cases. One-hundred-two isolates were grown in Columbia agar with 5% sheep blood (Becton Dickinson, Franklin Lakes, NJ, USA), but did not grow in staphylococcus selective agar (mannitol salt agar, Becton Dickinson) or enterococcus selective agar (Enterococcosel agar, Becton Dickinson). Forty-five of the 102 isolates were negative for the catalase test. Twenty-seven of the 45 isolates were identified as *S. canis*. Among the samples in which *S. canis* was detected, other organisms were detected in six specimens. Therefore, 27/546 (5%) of the gram-positive cocci-associated deep pyoderma detected were caused by *S. canis*.

Information on the dogs with *S. canis*-associated deep pyoderma is provided in Table 1. Among the 27 patients with *S. canis*-associated deep pyoderma, the samples were taken from abscesses in 10, followed by cellulitis in three. No preferred breed or sex of the affected dogs was found. By contrast, 15 of the 27 affected dogs were older than 10 years, with a mean ± standard deviation (SD) of 9.67 ± 4.58 years for the affected dogs. This age distribution is significantly older than the mean ± SD age distribution of dogs kept in Japan (4.71 ± 3.62 years; *p* < 0.001) [19]. Of the 16 cases with the status of antibiotic use reported, 10 were treated using quinolone antibiotics. Two of these cases were also treated with cephalexin (Table 1).

**Table 1:** Signalment information for dogs affected with deep pyoderma from which *Streptococcus canis* was isolated

Strain name	Breed	Sex^a^	Age (year)	Diagnostic name^b^	History of antibiotic administration^b^	Sequence typing	Clonal complex
P1	Pomeranian	F	0	Cellulitis	No administration	9	9
P2	Border collie	F	1	Panniculitis	No administration	46	2
P3	Poodle (toy)	FS	1	Abscess	Orbifloxacin	14	13
P4	Miniature schnauzer	FS	3	Bite wound	No administration	2	2
P5	Cardigan welsh corgi	FS	4	–	No administration	14	13
P6^c^	Shiba inu	FS	7	Bite wound	–	3	9
P7	Cocker spaniel	MC	8	Abscess	Cephalexin, enrofloxacin	27	13
P8	Shih tzu	M	8	–	Enrofloxacin	21	21
P9	Boston terrier	M	9	–	–	2	2
P10	Chihuahua	MC	9	Abscess	Orbifloxacin	9	9
P11^c^	Miniature schnauzer	F	9	Surgical site infection	–	9	9
P12	Beagle	F	9	Abscess	Orbifloxacin	3	9
P13	Labrador retriever	M	10	Abscess	–	46	2
P14	Chihuahua	M	10	Abscess	–	14	13
P15^c^	Yorkshire terrier	FS	11	Furunculosis	–	2	2
P16	Poodle (toy)	MC	11	Abscess	–	2	2
P17^c^	Labrador retriever	F	12	–	Enrofloxacin	19	5
P18	Golden retriever	M	12	Abscess	Cephalexin, orbifloxacin	14	13
P19	Border collie	FS	12	Cellulitis	–	14	13
P20	Miniature schnauzer	M	12	Cellulitis	–	56	33
P21^c^	French bulldog	M	13	–	–	3	9
P22^c^	Poodle (toy)	M	14	Abscess	Minocycline, fosfomycin	2	2
P23	Shiba inu	M	14	Abscess	Cephalexin	46	2
P24	Pomeranian	MC	15	–	Orbifloxacin	13	13
P25	Labrador retriever	M	15	Necrotizing fasciitis	No administration	41	55
P26	Chihuahua	MC	16	–	–	9	9
P27	Poodle (toy)	MC	16	–	Tylosin	48	9

^a^*MC* male castrated, *FS* female spayed. ^b^The “-” symbol indicates no data

^c^ Bacteria other than *S. canis* were also collected from specimens. P6, P15, P21 samples were also detected *Escherichia coli*. P11 and P17 samples were also detected *Corynebacterium* spp. and P22 sample also detected *Proteus mirabilis*

### Clinical features of dogs with sampling from the oral cavity

Twenty-six strains of *S. canis* were detected in samples from the oral cavity of 82 dogs with no clinical signs. Information about the dogs is provided in Supplementary Table 1. There were no significant differences in sex, age, or breed composition from the cases of deep pyoderma in which *S. canis* was isolated (*p* > 0.05).

### Phenotypic and genotypic properties of *S. canis* isolates

All isolates were β-hemolytic and carried the Lancefield group G polysaccharide. The API 20 Strep system identified all as *S. canis*. Among the 26 distinct biochemical profiles that were found with this system, 8 (*n* = 53 isolates, including the strain DSM 20715) corresponded to *S. canis* with a confidence of ≥99% according to the manufacturer’s instructions. All strains were detected following PCR amplification of the *S. canis ISR* and *SodA* genes.

### Distribution of clonal complexes (CCs) of *S. canis* from cases of deep pyoderma and oral samples

Multilocus sequence typing (MLST) analysis was performed on 53 strains of *S. canis*, 27 strains derived from dogs with deep pyoderma and 26 oral-derived controls. MLST analysis revealed a diverse collection, with nine CCs and two independent sequence typings (STs) (Table 2). Of the lesion-derived strains, 23/27 (85%) were classified in CC2, CC9, and CC13. The oral-derived strains were mainly CC2 (62%). CC9 was also detected in the oral cavity (12%), but CC13 was never detected in the oral-derived strains. *S. canis* from specimens in which multiple organisms were detected simultaneously were often CC2 and CC9 (5/6 specimens). We found no significant differences in the age, sex, API phenotype, or breed of diseased animals from which CC2, CC9, and CC13 were isolated.

**Table 2:** Comparison of the clonal backgrounds of strains colonizing either cases of deep pyoderma or oral cavity controls

Clonal complex (CC) or sequence type (ST) of colonizing strain	Case of deep pyoderma. n (%)	NC controls, n (%)
CC1	0 (0)	2 (7.7)
CC2	8 (29.6)	16 (61.5)
CC5	1 (3.7)	0 (0)
CC8	0 (0)	1 (3.8)
CC9	8 (29.6)	3 (11.5)
CC13	7 (25.9)	0 (0)
CC21	1 (3.7)	0 (0)
CC33	1 (3.7)	0 (0)
CC55	1 (3.7)	2 (7.7)
ST66	0 (0)	1 (3.8)
ST69	0 (0)	1 (3.8)

Singleton isolates that do not fall within a defined clonal complex are presented as per their multilocus sequence type

### Comparison of phylogenetic trees

**Fig. 1:**
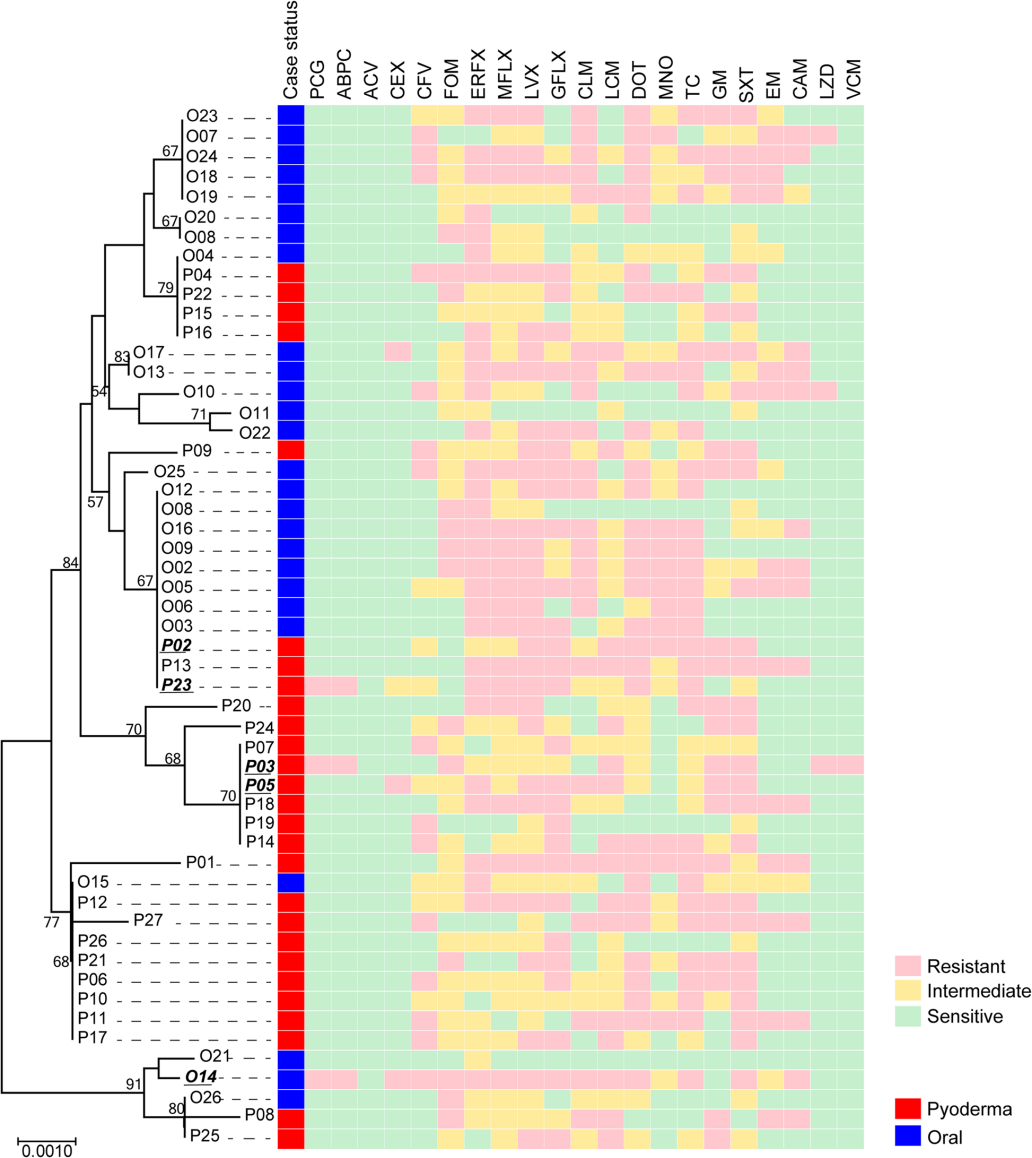
Antibiotic resistance profile of deep pyoderma cases and control isolates. A neighbor-joint tree of 53 isolates (27 cases of deep pyoderma, 26 oral controls) was built with multilocus sequence typing data. The strains indicated in bold italics produced a minimum inhibition concentration to penicillin, cephems, and anti-methicillin-resistant *Staphylococcus aureus* drugs, and β-lactamase activity. The status of each isolate is indicated by the colored cell (red, deep pyoderma; blue, oral control). Colored cells then indicate the presence of resistance to the antibiotic agent, namely penicillin G (PCG), amoxicillin (ABPC), amoxicillin/clavulanic acid (ACV), cephalexin (CEX), cefovecin (CFV), fosfomycin (FOM), enrofloxacin (ERFX), marbofloxacin (MFLX), levofloxacin (LVX), gatifloxacin (GFLX), clindamycin (CLM), lincomycin (LCM), doxycycline (DOT), minocycline (MNO), tetracycline (TC), gentamicin (GM), sulfamethoxazole and trimethoprim (SXT), erythromycin (EM), kanamycin (CAM), and the anti-methicillin-resistant *Staphylococcus aureus* (MRSA) drugs linezolid (LZD) and vancomycin (VCM) in a disk diffusion test (resistant, pink; intermediate, yellow; sensitive, green). Antibiotic resistance status was determined based on clinical and laboratory standards institute criteria

The seven sequence sequences used in the MLST analysis were combined for phylogenetic tree analysis (Fig. 1). A group of strains from CC13, a strain derived from deep pyoderma, and P20 strain, which is included in CC33, formed a cluster at a distance from the cluster containing other strains with oral origin.

### Distribution of antibiotic resistance phenotypes

From disk diffusion testing, among the isolates, the percentage of strains resistant to each antibiotic agent was not significantly different between strains from lesional or oral origin (*p* > 0.05) (Fig. 1, Supplementary Table 2). We found 6% of strains were resistant to penicillins (penicillin G (PCG), amoxicillin (ABPC)), but no strains were resistant to amoxicillin/clavulanic acid, which was formulated with β-lactamase inhibitors. By contrast, strains were resistant to quinolones (enrofloxacin (ERFX), marbofloxacin (MFLX), levofloxacin (LVX), and gatifloxacin (GFLV)) in the range of 38–59%. We also detected strains resistant to lincomycins (clindamycin (CLM), lincomycin (LCM)) and aminoglycosides (gentamicin (GM), sulfamethoxazole and trimethoprim (SXT)) in the range of 32–51%. Strains harbored resistance to tetracyclines (doxycycline (DOT), minocycline (MNO), tetracycline (TC)) in the range of 25–57%. Strains resistant to cephems (cephalexin (CEX), cefovecin (CFV)), macrolides (erythromycin (EM), kanamycin (CAM)), and fosfomycin (FOM) were detected in the range 6–28%. Strains resistant to anti-Methicillin-resistant *Staphylococcus aureus* (MRSA) drugs (linezolid (LZD), vancomycin (VCM)), which are not commonly used in veterinary medicine, were found in 2–6%. The number of strains resistant to three or more antibiotic classes (i.e., multidrug-resistant strains) was 37/53 (70%).

### Distribution of antibiotic resistance phenotypes

The phylogenetic tree and the distribution of antibiotic resistance phenotypes showed that resistance phenotype was evenly distributed in all clusters. The distribution of multidrug-resistant strains did not differ significantly among clusters (60–85%). Notably, the P03 strain resistant to penicillins, vancomycin, and linezolid, which formed lesion-specific clusters was classified as a CC13 strain in the phylogenetic tree (Fig. 1).

### Degree of minimum growth inhibition concentration (MIC)

We measured MICs of penicillins, cephems, and anti-MRSA drugs for P03, P23, O14, penicillin-resistant, and P02, P05, which was molecularly epidemiologically close to P03 and P23. Like the disk dilution results, P03, P23, and O14 were resistant to penicillins, but not to β-lactamase inhibitors. P03, P23, and O14 were resistant to some cephem antibiotics, but not others, depending on the drug. P03 also showed resistance to anti-MRSA drugs (Table 3).

**Table 3:** Minimum inhibitory concentration (MIC) of antibiotics against microorganisms

Antibiotics	CLSI Break point^**a**^			MIC (μg/ml)				
	S	I	R	P02	P03	P05	P23	O14
*Penicillins*								
Penicillin G	≤0.12	–	≥0.25	≤0.06	0.12	≤0.06	0.25	0.25
Ampicillin	≤0.25	–	≥0.5	≤0.25	2	≤0.25	0.5	0.5
*Penicillins/ β-lactamase inhibitors*								
Amoxicillin/clavulanate	≤2/1	4/2	≥8/4	≤0.25/0.12	1/0.5	≤0.25/0.12	≤0.25/0.12	≤0.25/0.12
*Cephems*								
Cefaclor	≤1	2	≥4	≤2	4	≤2	16	8
Cefazolin sodium	≤2	4	≥8	≤0.5	1	≤0.5	1	2
*Anti-MRSA drugs*								
Linezolid	≤2	–	≥4	2	4	2	2	2
Vancomycin	≤1	–	≥2	≤0.5	8	≤0.5	1	≤0.5

^a^Ampicillin and cefazolin sodium used Vet-08 criteria, while M-100 criteria were used for the remaining antibiotics. S, sensitive; I, Intermediate; R, Resistant. CLSI, Clinical & Laboratory Standards Institute

### Quantitative evaluation of β-lactamase activity in *S. canis*

**Fig. 2:**
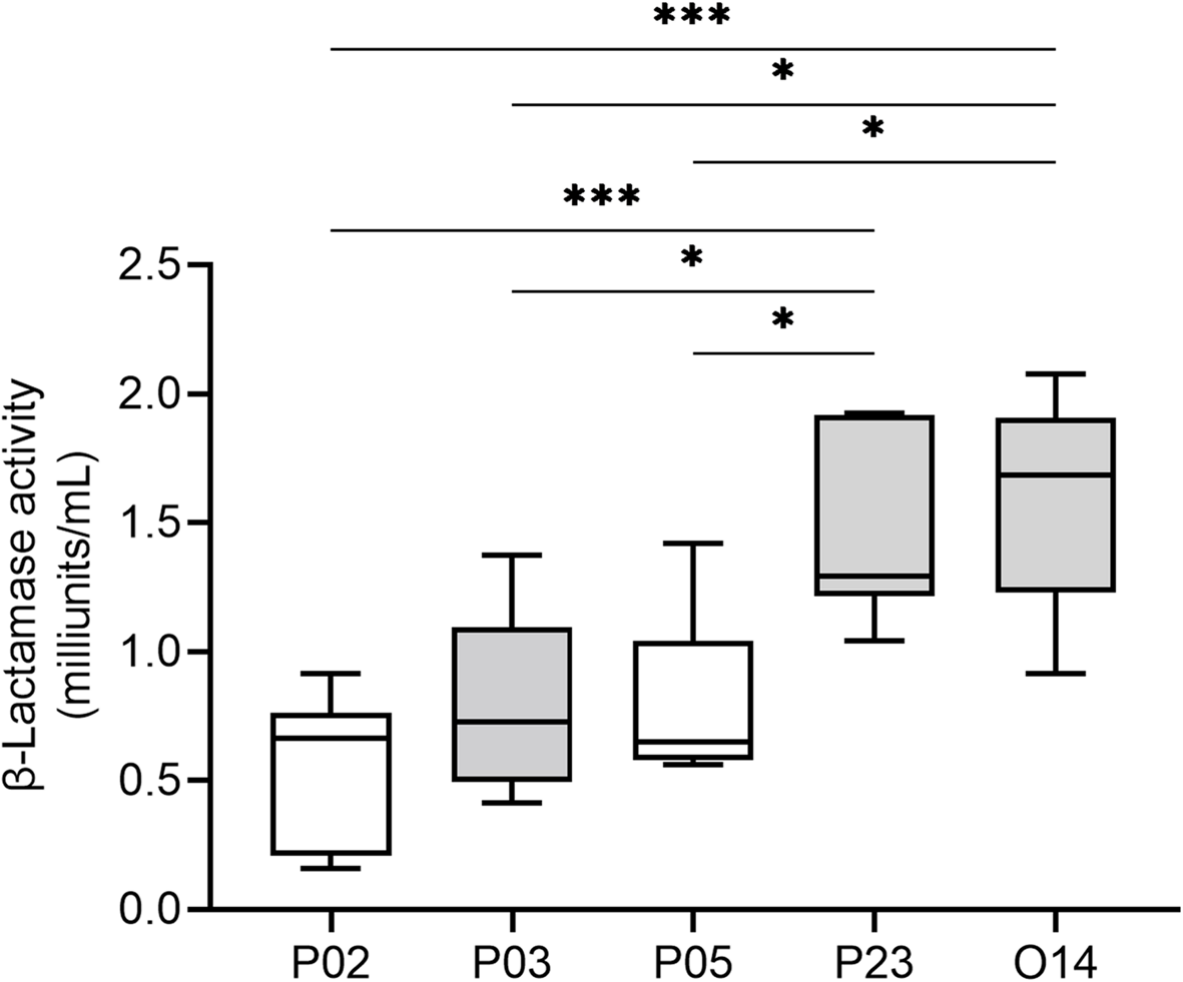
β-Lactamase activity profile in five strains of *S. canis.* Penicillin-resistant strains (P03, P23, 014) and penicillin-sensitive strains genetically close to P03 and P23 (P02, P05) were grown aerobically, and β-lactamase enzyme activity units were measured. P23 and O14 produced higher β-lactamase than the other strains (*p < 0.05, ***p < 0.005). No significant differences were observed among strains other than those bars shown in the figure (*p* > 0.05)

The five strains for measured MICs were also examined for β-lactamase activity (Fig. 2). Of the three strains that were resistant to penicillins, but not to amoxicillin/clavulanic acid, P23 and O14 strains had predominantly higher activity than the other strains (*p* < 0.05). By contrast, P03 possessed the same level of β-lactamase activity as penicillin-sensitive P02 and P05 (*p* > 0.05).

## Discussion

To our knowledge, our study is the first to analyze the antibiotic-resistant status and molecular typing of *S. canis* that causes canine deep pyoderma. Although *S. canis* showed aspects of an opportunistic infection, it may be that a highly pathogenic cluster with respect to CC13 has evolved. Penicillins are the most effective antibiotic agents for *S. canis*, but β-lactamase inhibitor-formulated penicillin should be used because some strains harbor β-lactamase.

*S. canis*-associated deep pyoderma was more likely to occur in older dogs. In humans, deep pyoderma caused by group G streptococci, including *S. canis*, has been shown to occur more frequently in the elderly with chronic diseases [20]. Although we were unable to follow up on the underlying disease of the canines with *S. canis*-associated deep pyoderma in this study, further analysis is required because immunocompromised hosts may be involved in the pathogenesis of deep pyoderma caused by *S. canis*. Erysipelas, a type of deep pyoderma, is known to show a bimodal pattern in young and old individuals [13]. Younger dogs have been reported to be susceptible to *S. canis* [21, 22]. We found no trend toward a higher incidence of pyoderma in young dogs, but this might be due to the small sample number used—further study to determine whether there is a tendency for young dogs to be more susceptible to *S. canis*-associated deep pyoderma is warranted.

Excessive use of inappropriate antibiotic agents may induce antibiotic resistance in *S. canis*-associated canine deep pyoderma. Similarly, the overuse of inappropriate antibiotics may induce drug resistance in *S. canis* in canine deep pyoderma. At present, Japanese veterinarians choose quinolones for 10/16 cases at first (62.5%), even though many quinolone-resistant strains were detected. In human medicine, multidrug-resistant *Streptococcus pyogenes* have increasingly been isolated, which is thought to be the result of the emergence of antibiotic-resistant strains from previously susceptible populations because of horizontal gene transmission and chromosomal point mutations caused by overuse of antibiotic agents [13, 23].

To treat *S. canis*-associated deep pyoderma, penicillins in combination with β-lactamase inhibitors are considered to be the most effective antibiotic agents [12, 13]. *Streptococcus* spp. carrying β-lactamases have also been found in other streptococci, and their increasing numbers in recent years are beginning to be a problem in human medicine [15, 23, 24]. We now have evidence that *S. canis* is beginning to show a trend similar to that of the streptococci. Note that the P03 strain showed resistance to penicillins despite that the absence of β-lactamase is likely to have another mechanism of resistance. For most staphylococci, the principal causative agents of deep pyoderma are gram-positive cocci [1], considered to be killed by penicillins containing a β-lactamase inhibitor [3, 25]. Therefore, when gram-positive cocci are detected in the drainage samples from deep pyoderma, penicillins with β-lactamase inhibitors should be used as the first line of treatment.

In the present study, CC2, CC9, and CC13 were isolated mainly from lesions of canine deep pyoderma. CC2 and CC9, which are also known as major isolates from animals and humans [26], were isolated from both the oral cavity and lesions of deep pyoderma in dogs. Furthermore, *S. canis* from specimens in which multiple organisms were detected simultaneously were often CC2 and CC9 (5/6 specimens). These data suggest that *S. canis*-associated deep pyoderma is an opportunistic infection, which is consistent with the prevailing theory [9] Unlike CC2 and CC9, CC13 was detected only in lesions, formed independent clusters in the phylogenetic tree, and was not a significant population among *S. canis* strains reported to date [26]. An analysis of *S. canis* causing ulcerative keratitis in dogs found that all were CC13 [27]. This suggests that CC13 may be a group of highly virulent strains prone to cause disease in dogs. Although there have been reports of toxic shock syndrome caused by *S. canis* in dogs [28, 29], few toxins from *S. canis* have been reported [30, 31]. Because CC13 is likely to have toxic factors that are pathogenic to dogs, analyses of this cluster will likely identify virulence factors such as the s erythrogenic toxins of *S. pyogenes*.

This paper has some limitations. Among 27 dogs with *S. canis*-associated deep pyoderma, 10 were diagnosed with abscesses, but these diagnoses were made independently by each physician, so it was not a uniform conclusion. In addition, the diagnostic criteria for erysipelas in the veterinary dermatology field are not clear to date [1]. It is also possible that some of the dogs were mixed with others who should have been diagnosed with erysipelas. A major obstacle to the diagnosis of erysipelas is the difficulty in identifying *S. canis* in clinical practice. Future studies on the usefulness of the Christie–Atkins–Munch-Peterson test and Antistreptolysin O antibody titers might make the detection of *S. canis* more convenient. Furthermore, the physician in charge of each sample transported it from each hospital in the study. In the case of *S. canis*-associated pyoderma, multiple bacteria were detected in six specimens, which suggests the possibility of contamination. We hope that future research will fill in the gaps in our analysis.

It is also a severe problem that the P03 strain, included in CC13, is resistant to the anti-MRSA drugs vancomycin and linezolid. Linezolid and vancomycin are among the last-resort antibiotic agents for treating multidrug-resistant gram-positive bacterial infections [32]. In enterococci, vancomycin-resistant forms are widespread in companion animals [33]. It would be worthwhile to confirm whether the vancomycin-resistant *S. canis* observed in this study was associated with the presence of the *van* gene from enterococci or with other mechanisms [32]. Because streptococci have been recognized as an antibiotic resistance reservoir in spreading resistance genes to major streptococcal pathogens in animals [34], the potential risks of disseminating resistant genes are worrisome. If this antibiotic resistance gene can be spread horizontally, it could lead to a refractory tendency and increase the mortality of affected patients when *S. canis* infects dogs and humans. Routine surveillance and the analysis of the resistant mechanisms should be strengthened.

## Conclusion

Although antibiotic-resistant strains were isolated at a high rate among *S. canis*, they can currently be treated with β-lactamase-inhibiting penicillins, CC13 may be a highly pathogenic cluster, and highly drug-resistant strains also exist. Further research on *S. canis* from both the veterinary clinical and bacteriological aspects will be necessary to identify these strains.

## Methods

### Bacteria isolated from canine deep pyoderma

Bacterial isolates were collected from the lesional skin of dogs in 719 clinical cases of canine deep pyoderma diagnosed by an attending veterinarian in 227 animal hospitals in Japan between August 2016 and March 2020 for diagnostic purposes. Sterile BBL culture swabs samples (Becton Dickinson), needle aspiration samples, and biopsy samples were used in this study. Before sample collection, we recommended physicians to wipe the skin surface once with alcohol-impregnated cotton swabs and wear surgical gloves. All samples were transported to the Vet Derm Tokyo laboratory at 4 °C after being placed in Stuart’s transport medium (Becton Dickinson). The plates were incubated in 5% CO_2_ at 35 °C for 48 h. We also incubated samples aerobically at 37 °C for 48 h on mannitol salt agar (Becton Dickinson) and enterococcosel agar (Becton Dickinson). One representative colony was isolated from each sample, and other pure cultures were cultured twice on Columbia agar with 5% sheep blood. The colonies were subjected to a Hacker variant Gram staining kit (Nissui Pharma, Tokyo, Japan). Strains that did not grow on mannitol salt agar but did grow on Columbia agar with 5% sheep blood were chosen, and the catalase activity of those gram-positive cocci was examined with 3% hydrogen peroxide (Kanto Chemical Co., Tokyo, Japan). The frozen isolates were stored at − 80 °C until used.

Information about the age, breed, sex, diagnostic name, and antibiotic used in the affected animal up to 3 weeks before sampling was collected from the physician in charge of each veterinary clinic. Some of the missing patient information was obtained by interviewing the veterinarian in charge by telephone.

### Bacteria isolated from the oral cavity of dogs

From March 1, 2021, to September 24, 2021, the oral cavity of 82 dogs with no clinical symptoms that came to the veterinary clinic for vaccination was swabbed with sterile swabs. The swabs were immediately streaked onto Columbia agar with 5% sheep blood and incubated in 5% CO_2_ at 35 °C for 48 h. One colony that showed β-hemolytic activity was isolated from each sample and cultured twice in pure culture on Columbia agar with 5% sheep blood. The colonies were stained with a Hacker variant Gram staining kit. For gram-positive cocci, catalase activity was examined using 3% hydrogen peroxide. The frozen isolates were stored at − 80 °C until used.

### Identification of *S. canis* species

*S. canis* was identified using a Lancefield latex agglutination test (Lancefield antigen A, B, C, D, F, and G) (Strept-LA NX Seiken kit, Denka, Tokyo, Japan), and API 20 Strep (bioMérieux, France). Bacterial DNA was extracted using an Easy DNA extraction kit version 2 (Kaneka, Tokyo, Japan) [35] and purified using a Monarch PCR and DNA cleanup kit (New England Bio Labs, Ipswich, MA, U.S.A.) [36] according to the manufacturer’s instructions, before being quantified using a NanoDrop 2000 spectrophotometer (Thermo Fisher). We also validated the accuracy of identification via PCR amplification of *S. canis ISR* and *SodA* genes [37, 38]. DSM20715 was used as a standard strain.

### MLST analysis

MLST analysis was performed on all the isolates included in the present study according to the protocol reported by Pinho et al. [26]. For sequence analysis, PCR products were transported and contracted for analysis by Eurofins Genomics Corporation (Tokyo, Japan). ST was determined using PubMLST (https://pubmlst.org/organisms/streptococcus-canis, accessed February 28, 2021).

STs were grouped into CCs, whereby related STs were classified as single locus variants differing in one housekeeping gene only. Based Upon Related Sequence Types analysis was performed on the MLST website to identify genetically related CCs. Trees were constructed and inference was performed using the neighbor-joining method after 500 iterations.

### Antibiotic susceptibility test

Disk diffusion testing was conducted using 21 different antibiotic agents. These were: PCG (disk content, 10 units, disk diffusion clinical breakpoints, S ≥ 24; R ≤ 23); ABPC (disk content, 10 μg, disk diffusion clinical breakpoints, S ≥ 24; R ≤ 23); ACV (disk content, 20/10 μg, disk diffusion clinical breakpoints, S ≥ 20; R ≤ 19); CEX (disk content, 30 μg, disk diffusion clinical breakpoints, S ≥ 18; I = 17–15; R ≤ 14); CFV (disk content, 30 μg, disk diffusion clinical breakpoints, S ≥ 24; I = 23–21; R ≤ 20); FOM (disk content, 50 μg, disk diffusion clinical breakpoints, S ≥ 16; I = 15–11; R ≤ 10); ERFX (disk content, 5 μg, disk diffusion clinical breakpoints, S ≥ 23; I = 22–17; R ≤ 16); MFLX (disk content, 5 μg, disk diffusion clinical breakpoints, S ≥ 18; I = 17–15; R ≤ 14); LVX (disk content, 5 μg, disk diffusion clinical breakpoints, S ≥ 17; I = 16–14; R ≤ 13); GFLX (disk content, 5 μg, disk diffusion clinical breakpoints, S ≥ 21; I = 20–18; R ≤ 17); CLM (disk content, 2 μg, disk diffusion clinical breakpoints, S ≥ 21; I = 20–15; R ≤ 14); LCM (disk content, 2 μg, disk diffusion clinical breakpoints, S ≥ 21; I = 20–17; R ≤ 16); DOT (disk content, 30 μg, disk diffusion clinical breakpoints, S ≥ 28; I = 27–25; R ≤ 24); MNO (disk content, 30 μg, disk diffusion clinical breakpoints, S ≥ 19; I = 18–15; R ≤ 14); TC (disk content, 30 μg, disk diffusion clinical breakpoints, S ≥ 23; I = 22–19; R ≤ 18); GM (disk content, 10 μg, disk diffusion clinical breakpoints, S ≥ 15; I = 14–13; R ≤ 16); SXT (disk content, 24/1 μg, disk diffusion clinical breakpoints, S ≥ 19; I = 18–16; R ≤ 15); EM (disk content, 15 μg, disk diffusion clinical breakpoints, S ≥ 21; I = 20–16; R ≤ 15); CAM (disk content, 15 μg, disk diffusion clinical breakpoints, S ≥ 21; I = 20–17; R ≤ 16); and the anti-MRSA drugs LZD (disk content, 30 μg, disk diffusion clinical breakpoints, S ≥ 21; R ≤ 20) and VCM (disk content, 30 μg, disk diffusion clinical breakpoints, S ≥ 17; R ≤ 16). We classified strains as susceptible, intermediate, and resistant against antibiotic agents using clinical and laboratory standards institute (CLSI) guidelines for Vet-08 and M-100-Ed31 [39, 40]. The strains resistant to three or more antibiotic classes were defined as multidrug-resistant strains referring to a previous report [41].

The MICs were determined by broth microdilution testing using a dry plate kit (Eiken, Tokyo, Japan) according to the manufacturer’s instructions [42].

*Staphylococcus pseudintermedius* isolated from canine superficial pyoderma samples was used for quality control [43]. Antibiotics were selected by suggested guidelines for using systemic antimicrobials in canine deep pyoderma [3], and drugs for which resistance can be a problem in the medical field, as well as drugs that are frequently used in Japanese veterinary medicine [44].

### β-Lactamase activity analysis

The β-lactamase activity was determined using a β-lactamase activity assay kit (Merck, Darmstadt, Germany) according to the manufacturer’s instructions [45]. The analyses were conducted in triplicate and performed twice.

### Ethics approval and consent to participate

The present study was conducted in compliance with the ARRIVE guidelines and applicable animal welfare regulations relating to the care and use of animals for scientific purposes. Informed consent was obtained from the owner of each participating dog. This experiment was approved by the Kyoto Veterinary Medical Center’s Animal Ethics Committee as having no ethical issues (approval number, KAMC-004).

### Statistical analysis

All analyses were conducted with Prism 9 (Prism version 9.3.1, GraphPad, San Diego, CA, USA). Data were expressed as mean ± SD and range. Chi-square tests were used to analyze the age of animals with *S. canis*-associated deep pyoderma and the percentage of resistant isolates. Kruskal–Wallis tests were used to compare the β-lactamase activity among five strains. All variable tests set *p* < 0.05 as statistically significant.

## Supplementary Information

**Additional file 1: Supplementary Table 1.** Signalment information of dogs with *S. canis* isolated from the oral cavity.

**Additional file 2: Supplementary Table 2.** Comparison of the antimicrobial resistance of strains colonizing either deep pyoderma or oral cavity controls.

## Data Availability

All data generated or analyzed during this study are included in this published article and its supplementary information files. The MLST datasets generated during the current study are available in the pub-MLST repository [https://pubmlst.org/], and the NCBI Repository (BioProject number: PRJNA885842) [https://www.ncbi.nlm.nih.gov/bioproject/].

## Abbreviations

ABPC: Amoxicillin
ACV: Amoxicillin/clavulanic acid
CC: Clonal complex
CEX: Cephalexin
CFV: Cefovecin
EM: Erythromycin
ERFX: Enrofloxacin
CAM: Kanamycin
CLM: Clindamycin
CLSI: Clinical and laboratory standards institute
DOT: Doxycycline
FOM: Fosfomycin
GFLX: Gatifloxacin
GM: Gentamicin
LCM: Lincomycin
LVX: Levofloxacin
LZD: Linezolid
MFLX: Marbofloxacin
MIC: Minimum inhibitory concentration
MRSA: Methicillin-resistant *Staphylococcus aureus*
MLST: Multilocus sequence typing
MNO: Minocycline
PCG: Penicillin G
SD: Standard deviation
ST: Sequence typing
SXT: Sulfamethoxazole and trimethoprim
TC: Tetracycline
VCM: Vancomycin

## References

[1] Miller WH, Griffin CE, Campbell KL, Muller GH, Scott DW (2013). Muller & Kirk's small animal dermatology.

[2] Loeffler A, Lloyd DH (2018). What has changed in canine pyoderma? A narrative review. Vet J.

[3] Beco L, Guaguère E, Lorente Méndez C, Noli C, Nuttall T, Vroom M (2013). Suggested guidelines for using systemic antimicrobials in bacterial skin infections: part 2-- antimicrobial choice, treatment regimens and compliance. Vet Record.

[4] Galpérine T, Cazorla C, Blanchard E, Boineau F, Ragnaud J-M, Neau D (2007). *Streptococcus canis* infections in humans: retrospective study of 54 patients. J Inf Secur.

[5] Sharma B, Srivastava M, Srivastava A, Singh R (2012). Canine streptococcal toxic shock syndrome associated with necrotizing fasciitis: an overview. Vet World.

[6] Lam MM, Clarridge JE, Young EJ, Mizuki S (2007). The other group G streptococcus: increased detection of *Streptococcus canis* ulcer infections in dog owners. J Clin Microbiol.

[7] Deviriese LA, Hommez J, Kilpper-balz R, Schileifer K (1986). *Streptococcus canis* sp. nov.: a species of group G streptococci from animals. Int J Syst Evol Microbiol.

[8] Janos D, Imre K, Herman V (2012). Research on frequency and characterization of *Streptococcus canis* strains isolated from dog and cat. Bull Univ Agric Sci Vet Med Cluj-Napoca Horticult.

[9] Ludwig C, de Jong A, Moyaert H, El Garch F, Janes R, Klein U (2016). Antimicrobial susceptibility monitoring of dermatological bacterial pathogens isolated from diseased dogs and cats across Europe (ComPath results). J Appl Microbiol.

[10] Ossiprandi MC, Cattabiani F, Bottarelli E (2001). Streptococcal flora of the oral cavity of dogs. Rivista di Igiene Microbiologia Epidemiologia.

[11] Taniyama D, Abe Y, Sakai T, Kikuchi T, Takahashi T (2017). Human case of bacteremia caused by *Streptococcus canis* sequence type 9 harboring the scm gene. IDCases.

[12] Sykes JE (2013). Canine and feline infectious diseases.

[13] Stevens DL, Bryant AE, Ferretti JJ, Stevens DL, Fischetti VA (2016). Impetigo, erysipelas and cellulitis. Streptococcus pyogenes: basic biology to clinical manifestations.

[14] Fukushima Y, Tsuyuki Y, Goto M, Yoshida H, Takahashi T (2019). Species identification of β-hemolytic streptococci from diseased companion animals and their antimicrobial resistance data in Japan (2017). Jpn J Infect Dis.

[15] von Specht M, García Gabarrot G, Mollerach M, Bonofiglio L, Gagetti P, Kaufman S (2021). Resistance to β-lactams in *Streptococcus pneumoniae*. Rev Argent Microbiol.

[16] Haenni M, Hourquet C, Saras E, Madec JY (2015). Genetic determinants of antimicrobial resistance in *Streptococcus canis* in France. J Glob Antimicrob Resist.

[17] Fukushima Y, Tsuyuki Y, Goto M, Yoshida H, Takahashi T (2020). Novel quinolone nonsusceptible *Streptococcus canis* strains with point mutations in quinolone resistance-determining regions and their related factors. Jpn J Infect Dis.

[18] Tsuyuki Y, Kurita G, Murata Y, Goto M, Takahashi T (2017). Identification of group G streptococcal isolates from companion animals in Japan and their antimicrobial resistance patterns. Jpn J Infect Dis.

[19] Holdings A (2021). White paper on household animal. Animal media.

[20] Fujiya Y, Hayakawa K, Gu Y, Yamamoto K, Mawatari M, Kutsuna S (2019). Age-related differences in clinical characteristics of invasive group G streptococcal infection: comparison with group a and group B streptococcal infections. PLoS One.

[21] Weingart C, Kershaw O, Kohn B, Rohwedder T (2022). Life-threatening acute neutrophilic vasculitis in a Shar-Pei puppy. Tierärztliche Praxis Ausgabe K: Kleintiere / Heimtiere.

[22] Lamm CG, Ferguson AC, Lehenbauer TW, Love BC (2010). Streptococcal infection in dogs: a retrospective study of 393 cases. Vet Pathol.

[23] Hakenbeck R, Grebe T, Zähner D, Stock JB (1999). Beta-lactam resistance in *Streptococcus pneumoniae*: penicillin-binding proteins and non-penicillin-binding proteins. Mol Microbiol.

[24] Bonofiglio L, Gagetti P, García Gabarrot G, Kaufman S, Mollerach M, Toresani I (2018). Susceptibility to β-lactams in β-hemolytic streptococci. Rev Argent Microbiol.

[25] Hillier A, Lloyd DH, Weese JS, Blondeau JM, Boothe D, Breitschwerdt E (2014). Guidelines for the diagnosis and antimicrobial therapy of canine superficial bacterial folliculitis (antimicrobial guidelines working group of the international society for companion animal infectious diseases). Vet Dermatol.

[26] Pinho MD, Foster G, Pomba C, Machado MP, Baily JL, Kuiken T (2019). *Streptococcus canis* are a single population infecting multiple animal hosts despite the diversity of the universally present M-like protein SCM. Front Microbiol.

[27] Enache AE, Mitchell C, Kafarnik C, Waller AS (2020). *Streptococcus canis* multilocus sequence typing in a case series of dogs with ulcerative keratitis. Vet Ophthalmol.

[28] Miller CW, Prescott JF, Mathews KA, Betschel SD, Yager JA, Guru V (1996). Streptococcal toxic shock syndrome in dogs. J Am Vet Med Assoc.

[29] DeWinter LM, Prescott JF (1999). Relatedness of *Streptococcus canis* from canine streptococcal toxic shock syndrome and necrotizing fasciitis. Can J Vet Res.

[30] Cornax I, Zulk J, Olson J, Fulde M, Nizet V, Patras KA (2021). Novel models of *Streptococcus canis* colonization and disease reveal modest contributions of M-like (SCM) protein. Microorganisms..

[31] Bergmann S, Eichhorn I, Kohler TP, Hammerschmidt S, Goldmann O, Rohde M (2017). SCM, the M protein of *Streptococcus canis* binds immunoglobulin G. Front Cell Infect Microbiol.

[32] Du F, Lv X, Duan D, Wang L, Huang J (2019). Characterization of a linezolid- and vancomycin-resistant *Streptococcus suis* isolate that harbors *optrA* and *vanG* operons. Front Microbiol.

[33] Wada Y, Irekeola AA, Engku Nur Syafirah EAR, Yusof W, Lih Huey L, Ladan Muhammad S (2021). Prevalence of vancomycin-resistant enterococcus (VRE) in companion animals: the first meta-analysis and systematic review. Antibiotics.

[34] Alves-Barroco C, Rivas-García L, Fernandes AR, Baptista PV (2020). Tackling multidrug resistance in streptococci - from novel biotherapeutic strategies to nanomedicines. Front Microbiol.

[35] Yoshida  M, Sano S, Chien J-Y, Fukano H, Suzuki M, Asakura T (2021). A novel DNA chromatography method to discriminate *Mycobacterium abscessus* subspecies and macrolide susceptibility. eBioMedicine.

[36] Fomenkov A, Vincze T, Mersha F, Roberts Richard J (2018). Complete genome sequence and methylome analysis of *bacillus caldolyticus* NEB414. Genome Announcements.

[37] Hassan AA, Khan IU, Abdulmawjood A, Lämmler C (2003). Development of PCR assays for detection of *Streptococcus canis*. FEMS Microbiol Lett.

[38] Hassan AA, Akineden O, Usleber E (2005). Identification of *Streptococcus canis* isolated from milk of dairy cows with subclinical mastitis. J Clin Microbiol.

[39] Institute CaLS (2018). Performance standards for antimicrobial disk and dilution susceptibility tests for bacteria isolated from animals.

[40] Clinical, Institute LS (2017). Performance standards for antimicrobial susceptibility testing.

[41] Magiorakos AP, Srinivasan A, Carey RB, Carmeli Y, Falagas ME, Giske CG (2012). Multidrug-resistant, extensively drug-resistant and pandrug-resistant bacteria: an international expert proposal for interim standard definitions for acquired resistance. Clin Microbiol Infect.

[42] Sasaki T, Kikuchi K, Tanaka Y, Takahashi N, Kamata S, Hiramatsu K (2007). Methicillin-resistant *staphylococcus pseudintermedius* in a veterinary teaching hospital. J Clin Microbiol.

[43] Iyori K, Shishikura T, Shimoike K, Minoshima K, Imanishi I, Toyoda Y (2021). Influence of hospital size on antimicrobial resistance and advantages of restricting antimicrobial use based on cumulative antibiograms in dogs with *staphylococcus pseudintermedius* infections in Japan. Vet Dermatol.

[44] Makita K, Sugahara N, Nakamura K, Matsuoka T, Sakai M, Tamura Y (2021). Current status of antimicrobial drug use in Japanese companion animal clinics and the factors associated with their use. Front Vet Sci.

[45] Abraham EP, Chain E (1940). An enzyme from bacteria able to destroy penicillin. Nature.

