# Supplementary material for: Antibiotic-resistant status and pathogenic clonal complex of canine Streptococcus canis-associated deep pyoderma
Source: BMC Vet Res. 2022 Nov 9;18:395. doi: 10.1186/s12917-022-03482-3 (PMC9644607; doi:10.1186/s12917-022-03482-3)
Supplement: Supplementary file 1 — Additional file 1: Supplementary Table 1. Signalment information of dogs with S. canis isolated from the oral cavity. [file 12917_2022_3482_MOESM1_ESM.docx]

**Supplementary Table 1. Signalment information of dogs with *S. canis* isolated from the oral cavity**

| **Strain name** | **Breed** | **Sex*** | **Age (years)** | **Sequence typing** | **Clonal complex** |
| --- | --- | --- | --- | --- | --- |
| O01 | Russell Terrier | M | 0 | 46 | 2 |
| O02 | Dachshund | MC | 3 | 8 | 8 |
| O03 | Dachshund | F | 4 | 46 | 2 |
| O04 | Cardigan Welsh Corgi | FS | 5 | 2 | 2 |
| O05 | Russell Terrier | MC | 6 | 46 | 2 |
| O06 | Shih Tzu | F | 6 | 46 | 2 |
| O07 | Chihuahua | M | 7 | 2 | 2 |
| O08 | Dachshund | M | 7 | 39 | 55 |
| O09 | Pug | FS | 8 | 46 | 2 |
| O10 | Basset Hound | FS | 8 | 2 | 2 |
| O11 | French Bulldog | FS | 9 | 1 | 1 |
| O12 | Poodle (Toy) | F | 9 | 46 | 2 |
| O13 | Mix | MC | 9 | 7 | 9 |
| O14 | Siberian Husky | F | 9 | 22 | 9 |
| O15 | Poodle (Toy) | FS | 9 | 66 | - |
| O16 | Poodle (Toy) | M | 10 | 46 | 2 |
| O17 | Chihuahua | MC | 11 | 7 | 2 |
| O18 | Pomeranian | MC | 11 | 7 | 2 |
| O19 | Cardigan Welsh Corgi | MC | 12 | 7 | 2 |
| O20 | Shih Tzu | FS | 12 | 9 | 9 |
| O21 | Akita | MC | 12 | 69 | 69 |
| O22 | Cardigan Welsh Corgi | M | 13 | 1 | 1 |
| O23 | Shiba Inu | FS | 13 | 7 | 2 |
| O24 | Border Collie | F | 14 | 7 | 2 |
| O25 | Yorkshire Terrier | MC | 15 | 7 | 2 |
| O26 | Yorkshire Terrier | FS | 15 | 41 | 55 |

***** MC, male castrated; FS, female spayed.
