# Supplementary material for: Antibiotic-resistant status and pathogenic clonal complex of canine Streptococcus canis-associated deep pyoderma
Source: BMC Vet Res. 2022 Nov 9;18:395. doi: 10.1186/s12917-022-03482-3 (PMC9644607; doi:10.1186/s12917-022-03482-3)
Supplement: Supplementary file 2 — Additional file 2: Supplementary Table 2. Comparison of the antimicrobial resistance of strains colonizing either deep pyoderma or oral cavity controls. [file 12917_2022_3482_MOESM2_ESM.docx]

**Supplementary Table 2. Comparison of the antimicrobial resistance of strains colonizing either deep pyoderma or oral cavity controls.** No significant difference was observed for any drugs (*p* > 0.05). Disk diffusion tests for penicillin G (PCG), amoxicillin (ABPC), amoxicillin/clavulanic acid (ACV), cephalexin (CEX), cefovecin (CFV), fosfomycin (FOM), enrofloxacin (ERFX), marbofloxacin (MFLX), levofloxacin (LVX), gatifloxacin (GFLX), clindamycin (CLM), lincomycin (LCM), doxycycline (DOT), minocycline (MNO), tetracycline (TC), gentamicin (GM), sulfamethoxazole and trimethoprim (ST), erythromycin (EM), kanamycin (CAM), and the anti-MRSA drugs linezolid (LZD) and vancomycin (VCM) were performed. Resistance status was determined based on CLSI criteria. Each percentage is written in parentheses.

|  |  | |  | N (%) of isolates | | | | | | | | | | | | | | | | | | | | |
| --- | --- | --- | --- | --- | --- | --- | --- | --- | --- | --- | --- | --- | --- | --- | --- | --- | --- | --- | --- | --- | --- | --- | --- | --- |
| Antibiotic: | | | | PCG | ABPC | ACV | CEX | CFV | FOM | ERFX | MFLX | LVX | GFLX | CLM | LCM | DOT | MNO | TC | GM | ST | EM | CAM | LZD | VCM |
|  | Case | | |  |  |  |  |  |  |  |  |  |  |  |  |  |  |  |  |  |  |  |  |  |
| Antibiotic susceptibility pattern | | S | | 25 (92.6) | 25 (92.6) | 27 (100) | 25 (92.6) | 12 (44.4) | 7 (25.9) | 7 (25.9) | 4 (14.8) | 0 (0) | 4 (14.8) | 10 (37.0) | 2 (7.4) | 6 (22.2) | 16 (59.3) | 4 (14.8) | 7 (25.9) | 1 (3.7) | 21 (77.8) | 21 (77.8) | 26 (96.3) | 26 (96.3) |
|  |  | I | | 0 (0) | 0 (0) | 0 (0) | 1 (3.7) | 6 (22.2) | 15 (55.6) | 11 (40.7) | 16 (59.3) | 12 (44.4) | 4 (14.8) | 11 (40.8) | 11 (40.8) | 8 (29.6) | 6 (22.2) | 10 (37.0) | 3 (11.1) | 8 (29.6) | 0 (0) | 0 (0) | 0 (0) | 0 (0) |
|  |  | R | | 2 (7.4) | 2 (7.4) | 0 (0) | 1 (3.7) | 9 (33.3) | 5 (18.5) | 9 (33.3) | 7 (25.9) | 15 (55.6) | 19 (70.4) | 6 (22.2) | 14 (51.8) | 13 (48.1) | 5 (18.5) | 13 (48.2) | 17 (63.0) | 18 (66.7) | 6 (22.2) | 6 (22.2) | 1 (3.7) | 1 (3.7) |
|  | Control | | | |  |  |  |  |  |  |  |  |  |  |  |  |  |  |  |  |  |  |  |  |
| Antibiotic susceptibility pattern | | S | | 25 (96.2) | 25 (96.2) | 26 (100) | 24 (92.3) | 17 (65.4) | 6 (23.1) | 1 (3.8) | 3 (11.5) | 3 (11.5) | 11 (42.3) | 5 (19.2) | 13 (50.0) | 5 (19.2) | 8 (30.7) | 7 (26.9) | 15 (57.7) | 7 (26.9) | 11 (42.3) | 15 (57.7) | 24 (92.3) | 26 (100) |
|  |  | I | | 0 (0) | 0 (0) | 0 (0) | 0 (0) | 3 (11.5) | 13 (50.0) | 4 (15.4) | 10 (38.5) | 7 (26.9) | 6 (23.1) | 4 (15.4) | 10 (38.5) | 4 (15.4) | 10 (38.6) | 2 (7.7) | 6 (23.1) | 10 (38.5) | 7 (26.9) | 2 (7.7) | 0 (0) | 0 (0) |
|  |  | R | | 1 (3.8) | 1 (3.8) | 0 (0) | 2 (7.7) | 6 (23.1) | 7 (26.9) | 21 (80.0) | 13 (50.0) | 16 (61.5) | 9 (34.6) | 17 (65.4) | 3 (11.5) | 17 (65.4) | 8 (30.7) | 17 (65.4) | 5 (19.2) | 9 (34.6) | 8 (30.8) | 9 (34.6) | 2 (7.7) | 0 (0) |
|  | Total | | |  |  |  |  |  |  |  |  |  |  |  |  |  |  |  |  |  |  |  |  |  |
| Antibiotic susceptibility pattern | | S | | 50 (94.3) | 50 (94.3) | 53 (100) | 49 (92.5) | 29 (54.7) | 13 (24.5) | 8 (15.1) | 7 (13.2) | 3 (5.7) | 15 (28.3) | 15 (28.3) | 15 (28.3) | 11 (20.8) | 24 (45.3) | 11 (20.8) | 22 (41.5) | 8 (15.1) | 32 (60.4) | 36 (67.9) | 50 (94.3) | 52 (98.1) |
|  |  | I | | 0 (0) | 0 (0) | 0 (0) | 1 (1.8) | 9 (17.0) | 28 (52.8) | 15 (28.3) | 26 (49.1) | 19 (35.8) | 10 (18.9) | 15 (28.3) | 21 (39.6) | 12 (22.6) | 16 (30.2) | 12 (22.6) | 9 (17.0) | 18 (34.0) | 7 (13.2) | 2 (3.8) | 0 (0) | 0 (0) |
|  |  | R | | 3 (5.7) | 3 (5.7) | 0 (0) | 3 (5.7) | 15 (28.3) | 12 (22.7) | 30 (56.6) | 20 (37.7) | 31 (58.5) | 28 (52.8) | 23 (43.4) | 17 (32.1) | 30 (56.6) | 13 (24.5) | 30 (56.6) | 22 (41.5) | 27 (51.0) | 14 (26.4) | 15 (28.3) | 3 (5.7) | 1 (1.9) |
